# My paediatric infectious diseases journey

**DOI:** 10.4102/sajid.v37i2.381

**Published:** 2022-02-14

**Authors:** Raziya A. Bobat

**Affiliations:** 1 Enhancing Care Foundation, Durban, South Africa; 2 Department of Paediatrics and Child Health, Faculty of Health Sciences, University of KwaZulu-Natal, Durban, South Africa

It was the 1st of June 1989 and my first day back at work after a 6-month maternity break. I had barely settled down at my desk when my Head of Department, Prof. Jerry Coovadia, walked into my office and said: ‘Raziya, we have a problem. We have HIV’. And so began my 30-year journey in Paediatric Infectious Diseases (ID). It was sometimes smooth, at times bumpy and, occasionally, a roller-coaster ride. But, there was never a dull moment. I faced countless challenges and obstacles, but the smiling faces of the many children I cared for over the years made everything worthwhile.

Paediatric ID was not a recognised subspecialty until 2006, when the first group of clinicians were registered as Paediatric ID Specialists. Throughout my 30 years in Paediatric ID, the bulk of patients I cared for were children with HIV infection and tuberculosis (TB). It was completely new, and we learnt as we went along. We had no diagnostic tests (not even an ELISA) and had to rely on clinical judgement and ancillary tests (e.g. an elevated globulin level) that assisted in making a diagnosis.

In 1992, I established the Paediatric HIV Clinic, initially for the follow up of infants enrolled in my doctoral study.^1^ We had little to offer apart from a sympathetic ear for the mothers and multivitamin syrup for the infants. Later, co-trimoxazole was added to their care. We started antiretroviral therapy among children whose parents could afford it in 2002. Those were exciting times, as we finally had something to offer the children.

This clinic was one of the first in South Africa. Today it has one of the largest number of children and adolescents receiving ART. I was fortunate to have had the opportunity to gain experience in Paediatric HIV care, initially for a year at St Mary’s Hospital in London, followed by a scholarship that allowed me to spend a month at the Baylor Childrens’ Institute in Houston.

In 2009, my colleague, Dr Mo Archary, received his registration and the Paediatric ID Unit was established. We faced many challenges, including the lack of support, diagnostic tests, space and funding. We eventually created a dedicated area in the wards at King Edward VIII Hospital (KEH) in Durban, and the unit grew from strength to strength. We worked closely with colleagues from microbiology and virology, trying to provide the best service to our patients. Over the years, we trained several ID subspecialists and provided training to doctors from abroad.

My interest in research was ignited by Prof. Jerry Coovadia, who was my Doctor of Philosophy (PhD) supervisor and mentor. Together we published the first article on the clinical and laboratory features of children with HIV infection at KEH.^2^ He encouraged me to pursue a PhD and introduced me to two amazing colleagues: Prof. Zena Stein and Prof. Mervyn Susser from Columbia University, who assisted in drafting my protocol.

In 2003, I was approached by the paediatric AIDS Clinical Trials Group to set up a paediatric HIV research unit funded by the Division of Aids (DAIDS) (National Institute of Allergy and Infectious Diseases). This partnership lasted for 12 years and led to participation in many international trials.

In 2005, the management of KEH granted permission to set up a mobile parkhome clinic on the hospital’s grounds, specifically for research into paediatric HIV. The parkhome clinic continues to participate in multiple studies. It is a legacy that I am certainly proud of.

I had developed several HIV teaching and training programmes for students, doctors and nurses. In the early 2000s, I was one of a multidisciplinary team that provided training in HIV to staff across KZN under the auspices of the KZN Department of Health and with the support of the Red Cross Flying Division. The enthusiastic staff greeted us wherever we went, as HIV was new to everyone.

I have been a member of many national guideline committees and a founding member of the South African Society for Paediatric ID. I was also part of the team that successfully applied for the NIH Fogarty Medical Education Partnership Initiative grant, which allowed us to establish an HIV Diploma Programme that trained many doctors, nurses and pharmacists.

Infectious Diseases certainly is a specialty that I would encourage young doctors to follow. There is always something new, something unusual, to challenge you! Therefore, ID should be seen as cutting across all subspecialty, as a unit that supports all the other disciplines in caring for children with typical and unusual infections.

In December 2019, I officially retired from my position as Associate Professor/Principal Specialist and Head of the Paediatric ID Unit, but remain an honorary staff member. Currently, I work with a research organisation, Enhancing Care Foundation, on clinical trials in COVID-19 vaccines and therapeutics.

My career swansong was publishing the book ‘*HIV Infection in children and adolescents*’, released in March 2020.^3^
